# Analysis of the thermal insulation performance of cement with waste glass powder in geothermal well

**DOI:** 10.1038/s41598-024-67546-0

**Published:** 2024-08-09

**Authors:** Ying Ji, Li Song, Qianqian Sha, Gang Zhu, Yuze Xue, Tinghui Zhang, Shuai Fan

**Affiliations:** 1 College of Materials Science and Engineering, Xi’an University of Architecture and Technology, Xi’an, 710055 Shaanxi China; 2 China Building Materials Industry Construction Xi’an Engineering Limited Company, Xi’an, 710075 Shaanxi China; 3 Key Laboratory of Coal Resources Exploration and Comprehensive Utilization, Ministry of Natural Resources, Xi’an, 710021 Shaanxi China; 4 Shaanxi Coal Geology Group Co., Ltd., Xi’an, 710021 Shaanxi China

**Keywords:** Composites, Mechanical properties

## Abstract

To improve the heat extraction efficiency from the wellbore fluids to the stratum in the geothermal well, thermal insulation cement, which contains of waste glass powder as a heat-insulating material, is proposed to apply in geothermal well’s middle and upper sections in the paper. Effect of such glass powers on mechanic and thermal property of thermal insulation cement was then investigated. Various tests were carried out to measure compressive strength, thermal conductivity, microstructure porosity etc. parameters of the thermal insulation cement. Results showed that the waste glass powder would enhance its the compressive strength and improve its the thermal insulation performance. Correlation study between contents of the added waste glass powder in geothermal cements and its mechanic and thermal property was conducted. It was found that thermal insulation cement exhibited its optimum performance when the added content of glass powers was 20% in weight. Analysis of the microstructure porosity with SEM found that the pores in thermal insulation cement with added waste glass powders were mostly closed, tiny and even, and therefore contributed to the compressive strength of the thermal insulation cement; such pores would be also beneficial to improving its thermal insulation performance.

## Introduction

Recently, there has been a steady increase in the utilization of fossil fuels, leading to worsening environmental pollution^1^.To address this issue while ensuring economic progress, geothermal energy has emerged as a clean and sustainable alternative. Medium and low temperature geothermal resources hold great promise for the development of thermal energy.

Medium and low temperature geothermal resources use a geothermal coaxial system for heat extraction without water extraction. The geothermal coaxial systems are characterized by a large heat transfer area, high thermal power, and low construction costs^2^. In geothermal energy production, the wellhead temperature is a crucial parameter for the rational design of thermal extraction. As a heat transfer medium from the fluid in the wellbore to stratum, the heat transfer capacity of cement is a significant factor that influences the wellhead temperature. Heat loss is currently the biggest challenge for geothermal coaxial systems^3^.

In the middle and upper sections of the geothermal well, temperature of the geothermal fluids in the wellbore is higher than of the stratum^4^. Heat energy is transferred from the geothermal fluids to the stratum, causing heat loss and resulting in lower wellhead temperatures^5–8^. The outlet temperature of geothermal fluids is a significant basis for determining the applications of geothermal energy^9^. Heat loss will reduce the application rate of geothermal energy and increase the costs of geothermal production. Therefore, it is necessary to reduce the thermal conductivity of the cement, to minimize the heat transfer from the geothermal fluids to the stratum^10^, to lower the heat loss and improve the heat extraction efficiency of the middle and upper sections of the geothermal well^11,12^.

At present, some scholars have proved that the thermal conductivity of the concrete is reduced, which prepared by mixing a certain amount of fly ash^13^, rice husk ash^14^, metakaolin and Silica fume^15^, foams and hollow glass beads^16^ instead of a part of cement. These thermal insulation materials contain many SiO_2_ and Al_2_O_3_, volcanic ash effect can occur with the cement to improve the thermal insulation properties of cement. However, most of the traditional thermal insulation materials are lightweight and porous, which only improve the thermal insulation properties of cement while neglecting its mechanical properties. Waste glass is rich in silica, has a volcanic ash effect and can be used as an active admixture in cement, which is a type of non-degradable waste that accumulates in large quantities in landfills, causing serious soil and water pollution^17^.

Some scholars have discovered that adding discarded glass to concrete and mortar can enhance the performance of concrete, and utilizing waste glass as a replacement for cement is more effective than as a fine aggregate^18–20^. In addition, glass powder can reduce the likelihood of concrete corrosion^21^.Some authors^22–24^ pointed out that the volcanic ash effect of waste glass powder results in cement that displaying abundant hydration products, along with reduced micro cracks increased later strength. Furthermore, some scholars^25,26^ found that finely ground glass powder can reduce the porosity of the concrete better while increasing its compressive strength. Fine glass powder is also easier to produce the volcanic ash effect because of its larger specific surface area, and the volcanic activity of glass powder strengthens as its fineness decreases^27–29^.

Some scholars have studied the effect of waste glass powder on cement durability, that waste glass powder can not only increase the compressive strength of cement, but also improve the resistance to chloride ion permeability^30^, the chloride ion penetration, the alkali silica reaction (ASR), and the sulfate resistance^31^ and the acid corrosion inhibition potential^32^. Prior research showed that the use of waste glass in place of natural aggregates results in a significant reduction in the thermal conductivity of the mortar^33,34^. Yuwadee et al. found that the thermal conductivity of mortar prepared using automotive glass instead of sand was reduced by 63%^35^. Although previous researchers have done many studies on the mechanical properties and durability of mortar and concrete by replacing part of the cement with waste glass powder, and the effect of replacing sand with waste glass powder on the thermal conductivity of mortar has also been studied. However, the effect of replacing part of the cement with waste glass powder on the thermal conductivity of cement paste is still not clear. The thermal conductivity of cement in geothermal wells is of great engineering significance for the effect of heat loss of cement in the process of geothermal energy extraction.

Laminated glass and flat glass are fabricated through high-temperature calcination, resulting in their remarkable insulating capabilities. Glass possesses a condensed porous structure, with the primary constituent being SiO_2_, which has good thermal stability that can keep the long-term stability of cement in geothermal environments. The potential impacts of waste glass powder as a thermal insulation material incorporating into cement include can elevate the density of cement, the mechanical properties^36^, and thermal insulation performance, thereby enabling it to effectively function in intricate geothermal environments. In addition, the use of waste glass powder to prepare thermal insulation cement not only reduces heat transfer in the thermal insulating section of geothermal wells, lower heat loss, and improves the extraction rate and sustainability of geothermal energy, but also be helpful for mitigating the pressure of waste accumulation, saving the cost of cementing, and promoting the recycling economy, which provides a promising solution to reduce the heat loss of geothermal wells.

By substituting 0–20% waste glass powder for oil well cement in the preparation of thermal insulation cements, further research and testing are needed to accurately assess its potential impact. This includes evaluating the thermal conductivity, mechanical properties, porosity, and thickening time of thermal insulation cements with waste glass powder. The microscopic effects of waste glass powder on the cement paste were analyzed by X-ray diffraction analysis (XRD), thermogravimetric analysis, porosity, and SEM scan. The study can supply theoretical guidance to improve the thermal insulation performance of geothermal well cement.

## Materials and methods

### Experimental materials

**Figure 1:**
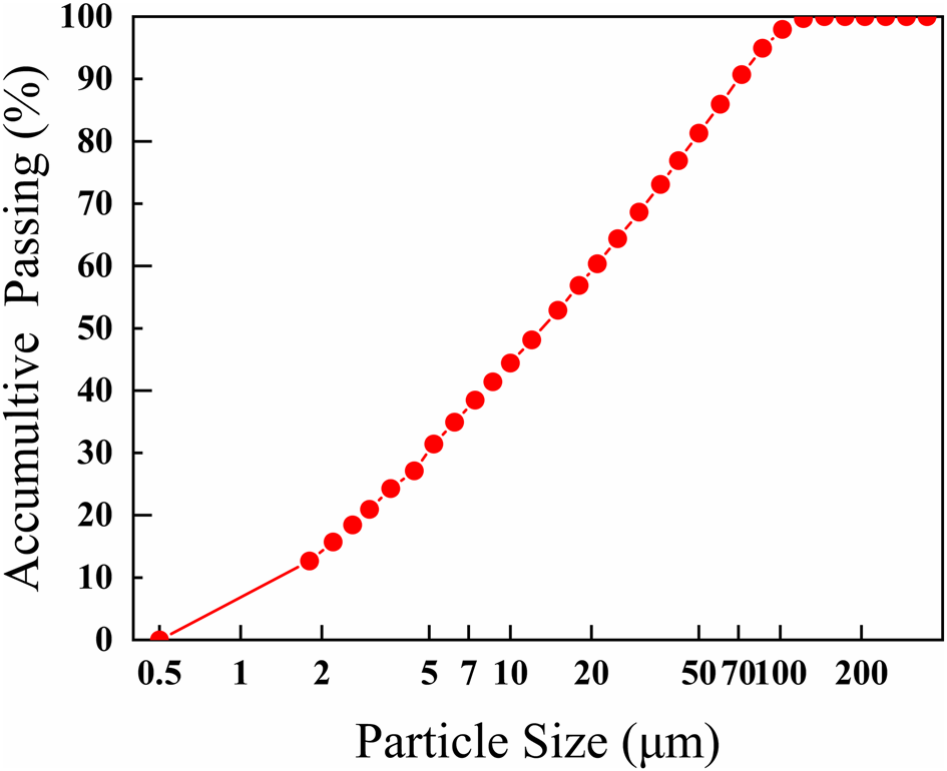
Distribution of waste glass powder’s particle sizes.

**Figure 2:**
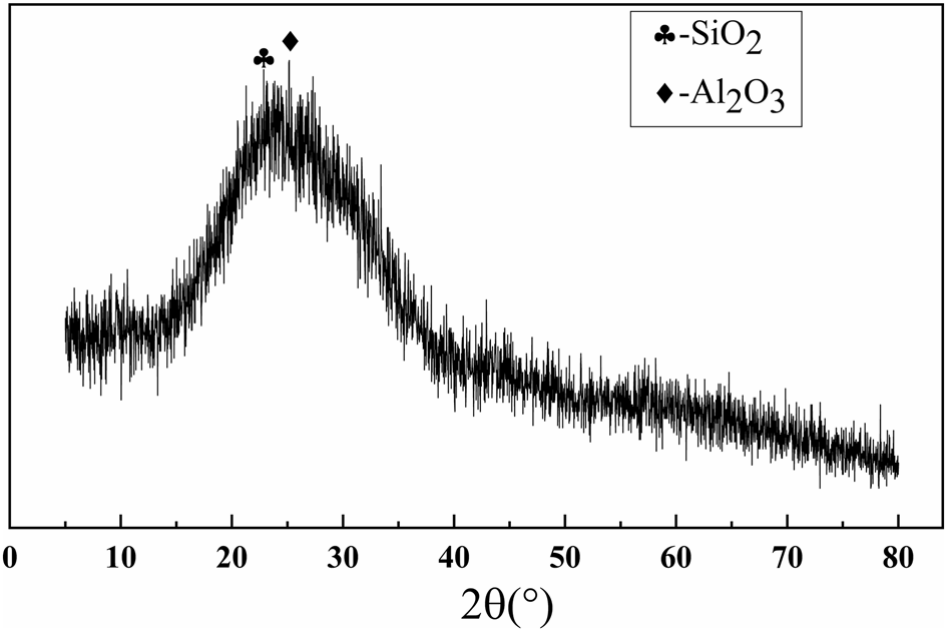
XRD pattern of waste glass powder.

Waste glass powder is mainly for waste laminated glass and flat glass, using ball mills and vibration mills to pulverize the waste glass and pass through a 200-mesh sieve screen. Figure 1 introduces the particle sizes distribution of waste glass powder, the particle size is mainly distributed between 0.5 and 72 μm. Table 1 shows the chemical composition of waste glass powder. The XRD diffraction pattern of waste glass powder is shown in Fig. 2. From the graph and Table 1, the primary composition of is SiO_2_, with a content of 70.02%.

**Table 1:** Chemical composition of waste glass powder.

Na_2_O	Al_2_O_3_	SiO_2_	Fe_2_O_3_	CaO	SO_3_	MgO
13.81	0.98	70.02	0.33	9.92	0.30	4.08

**Figure 3:**
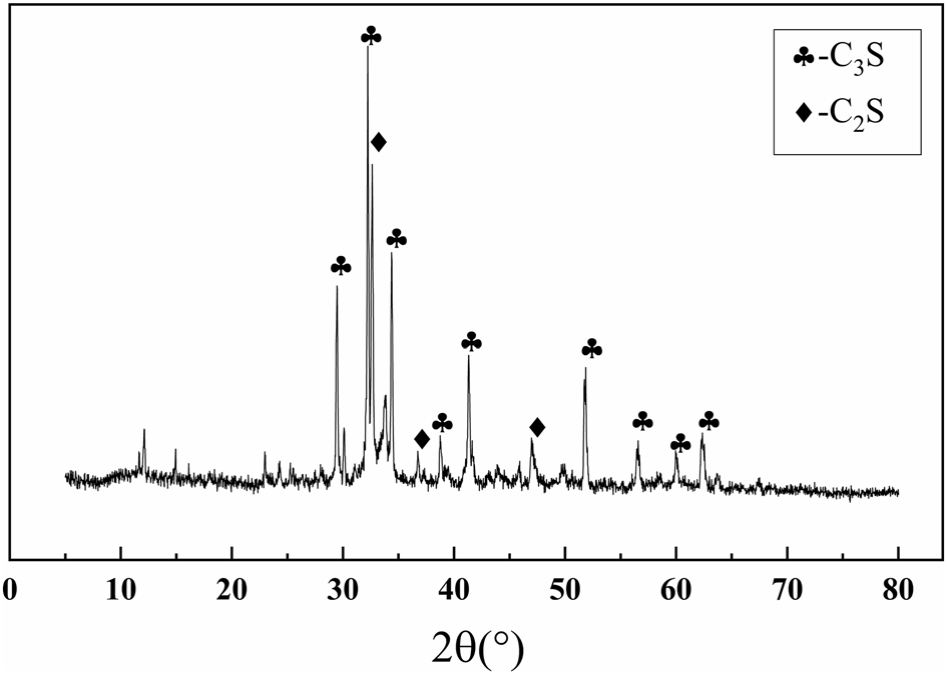
XRD pattern of oil well cement.

The chemical composition of G-grade oil well cement is shown in Table 2, the content of C_3_S is 54.10%, and the physical performance is shown in Table 3, the water–cement ratio was 0.44, and the 8 h compressive strength of oil well cement was 4.3 MPa at 38 °C and 15.1 MPa at 60 °C. The XRD diffraction pattern of the oil well cement as shown in Fig. 3, reveals its main phase composition to be C_3_S and C_2_S.

**Table 2:** Chemical composition of cement.

Chemical composition	MgO	SO3	C3S	C2S	C3A	C4AF + 2C3A	Burn vector	Insoluble substances	Total alkali content
Content (%)	3.10	1.87	54.10	16.48	2.00	19.86	1.12	0.56	0.56

**Table 3:** Physical performance of cement.

Density (g/cm^3^)	Specific surface area (m^2^/kg)	Water–cement ratio	35.6 MPa 52 °C		8 h compressive strength (MPa)	
			15–30 min maximum consistency (BC)	Thickening time (min)	38 °C	60 °C
3.18	349	0.44	13.5	109	4.3	15.1

Water reducing agent, 540P poly carboxylic acid, is white powder..Retarder, sodium gluconate, is a white transparent particle.

### Experimental method

#### Preparation of cement paste

Referring to the “Oil Well Cement” GB/T 10238-2015, preparing the thermal insulation cement, curing the cement paste at 38 °C and 60 °C under atmospheric pressure. Table 4 can be referred to prepare the thermal insulation cement slurry. The amount of waste glass powder (0%, 5%, 10%, 15%, 20%) was weighed, then cement (100%, 95%, 90%, 85%, 80%) was added until the weight of the two masses was 792.00 g. The weighed composition was mixed well without water.

**Table 4:** Cement paste ratio.

Curing temperature /°C	Number	Waste glass powder content/%	Quality of water/g	Quality of waste glass powder/g	Quality of oil well cement/g
38	G0	0	349.00	0	792.00
	G1	5	349.00	39.60	752.40
	G2	10	349.00	79.20	712.80
	G3	15	349.00	118.80	673.20
	G4	20	349.00	158.40	633.60
60	W0	0	349.00	0	792.00
	W1	5	349.00	39.60	752.40
	W2	10	349.00	79.20	712.80
	W3	15	349.00	118.80	673.20
	W4	20	349.00	158.40	633.60

Specifically, the weighed water was first poured into the slurry cup of the stirrer, to be stirred at 4000 r/min, the stirrer was started, and the mixed waste glass powder and cement were poured into the slurry cup with water within 15 s, the lid of the slurry cup was put on, to be continuously stirred at 12,000 r/min within 35 s. After the above steps, the cement slurry preparation was completed. The prepared cement slurry was poured to one-half of the depth of the test mold, and each test mold was vibrated 27 times using a stainless-steel rod with a diameter of 6 mm. The remaining slurry was stirred to prevent the slurry from segregating. The remaining cement paste was then poured into the test mold until it overflowed, and then evenly pounded 27 times, using a ruler to scrape off the excess paste from the upper part of the test mold. Cover the top of the test mold with a clean and dry cover.

#### Performance tests of cement paste

1. Compressive strength. After pouring the prepared cement slurry into (50 mm × 50 mm × 50 mm) copper molds within 5 min, the molds were demolded after 1 d of maintenance in a water bath at 38 °C and 60 °C, respectively. The YAW-300 was used to measure the compressive strength, the pressurization rate is 1.2 kN/s and the average value of four sets of data was calculated.
2. Thermal conductivity test. The prepared cement slurry was poured into (50 mm × 50 mm × 10 mm) copper molds within 2 min, and cured at room temperature for 1 d. After removing the molds, the paste was transferred to a curing box at 20 °C and 95% humidity for 28 d. The bumps on the surface of the thermal conductivity test block were smoothed with 80-mesh, 140-mesh, and 400-mesh sandpaper, respectively, for evaluating the coefficient of thermal conductivity. The TC 3100 thermal conductivity meter (employing the transient plane source method) was used to evaluate the thermal conductivity of cement paste, and the testing temperatures were 30 °C, 60 °C and 90 °C, respectively.
3. Thickening time. The thickening curve of the cement slurry was evaluated using OWC-2250B atmospheric pressure thickening instrument produced by Shenyang Petroleum Instrument Research Institute. The prepared cement slurry was poured into the pressurized thickener pulp cup and quickly sealed within 20 s. After placing the pulp cup, the circulating temperature of the pressurized thickener was set to 52 °C and the circulating pressure to 35 MPa. The pressure was manually adjusted to keep the temperature within the range of ± 1.0 °C of the set value and the pressure within the range of ± 0.7 MPa of the set value. The initial consistency and the maximum consistency within 30 min were recorded until the consistency of the samples reached 100 BC, and the graphs of the changes in temperature, pressure, and consistency during the thickening process were derived at the end of the experiment.

#### Micro tests

The cement samples after measuring the compressive strength were crushed with a hammer and the sample fragments were soaked in anhydrous ethanol for more than 7 d, dried at 80 °C for 24 h and then ground with a vibrating mill for 30 s for microscopic testing.

1. XRD: It was used to analyze the effect of incorporating waste glass powder on the hydration products of cement. The D/MAX 2200 X-ray diffraction analysis technology produced by Rikagu Corporation of Japan. Test parameters: Cu Kα target, tube voltage and current 40 kV, 40 mA, power 1.6 KW, scanning angle range of 5° ~ 80°. The scanning speed is 10°/min.
2. TG-DTG: It was used to analyze the hydration process, and the thermal stability of thermal insulation cement under air atmosphere. German Netzsch TG 209 F1 thermal analyzer. The heating rate is 10° C/min. The test temperature is 30–800 °C, the heating rate is 10° C/min, and the test environment is air.
3. Porosity: It was used to analyze the effect of waste glass powder on cement porosity. The Micromeritics Auto Pore IV 9500 mercury intrusion meters from the United States. The testing aperture range is 0.005–800 µm.
4. SEM: Thin slices of about 1 cm × 1 cm in size and flat on both sides were selected before the sample fragments were pulverized for SEM scanning electron microscope testing to observe the micro-morphological changes in the thermal insulation cement and to analyze the effect of the waste glass powder on the microstructure of the cement. The German ZEISS Sigma300 scanning electron microscope. Magnification range: 25–200,000 times, Resolution: 1.2 nm, Tested on thin sheet cement samples at 20 °C, Magnification: 5 KX and 10 KX.

## Results and discussion

### Compressive strength

**Figure 4:**
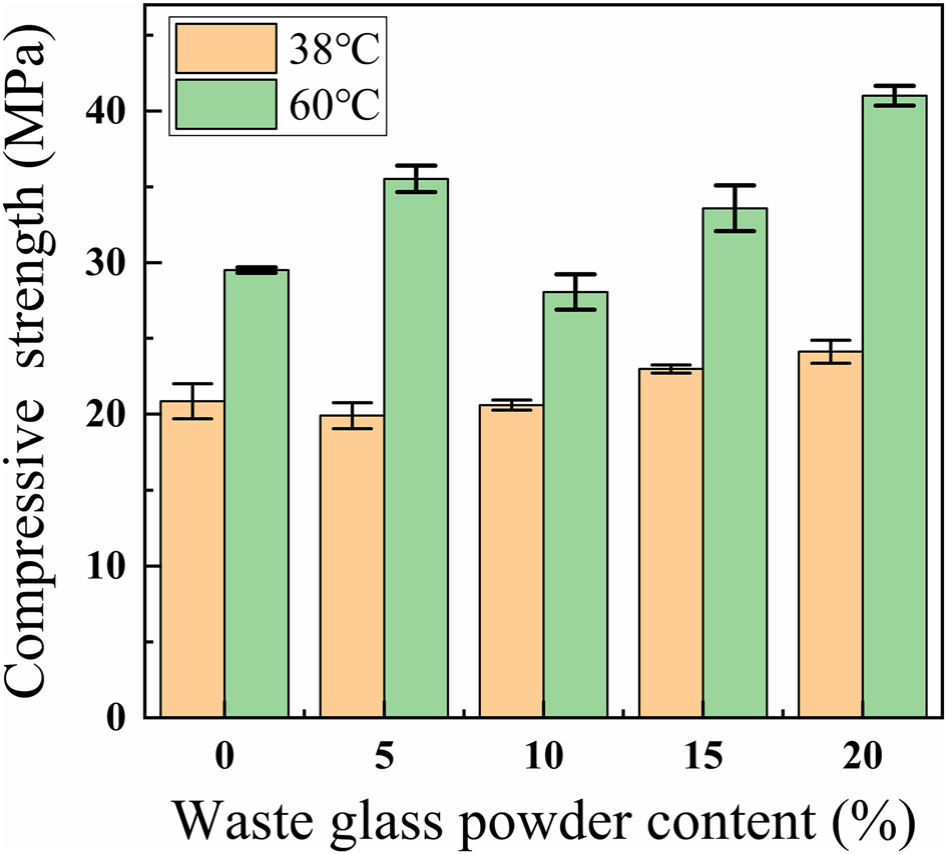
Compressive strength of cement paste with waste glass powder.

The test results from Fig. 4 manifested that adding waste glass powder boosted the compressive strength of the cement paste. The compressive strength of G0 and G4 were 20.87 MPa and 24.22 MPa, respectively, G4 with a growth rate of 16.10%. The compressive strength of W0 and W4 were 29.48 MPa and 41 MPa respectively, W4 with a growth rate of 39.08%.The main reason for the increase of compressive strength of waste glass powder cement is that the SiO_2_ in waste glass powder reacts with the substances in the cement to generate C–S–H gel that can provide compressive strength^37^, and the filling effect of waste glass powder as a fine powder on the cement voids, which increases the denseness of the cement paste.

This is contrary to the results of others study^38^, which is due to the small particle size of waste glass powder, which is more likely to occur in the cement paste with the microaggregate filling effect and the volcanic ash effect, reducing the porosity of the cement paste, also, this was related to the glass type and source.

The compressive strength of 60 °C insulation cement is greater than 38 °C, this is because of the f higher temperatures to promote the second hydration about the waste glass powder and the cement, to generate denser C–S–H gel, increase the densification of the cement paste, improve the compressive strength of cement^39^.

## Thermal conductivity

**Figure 5:**
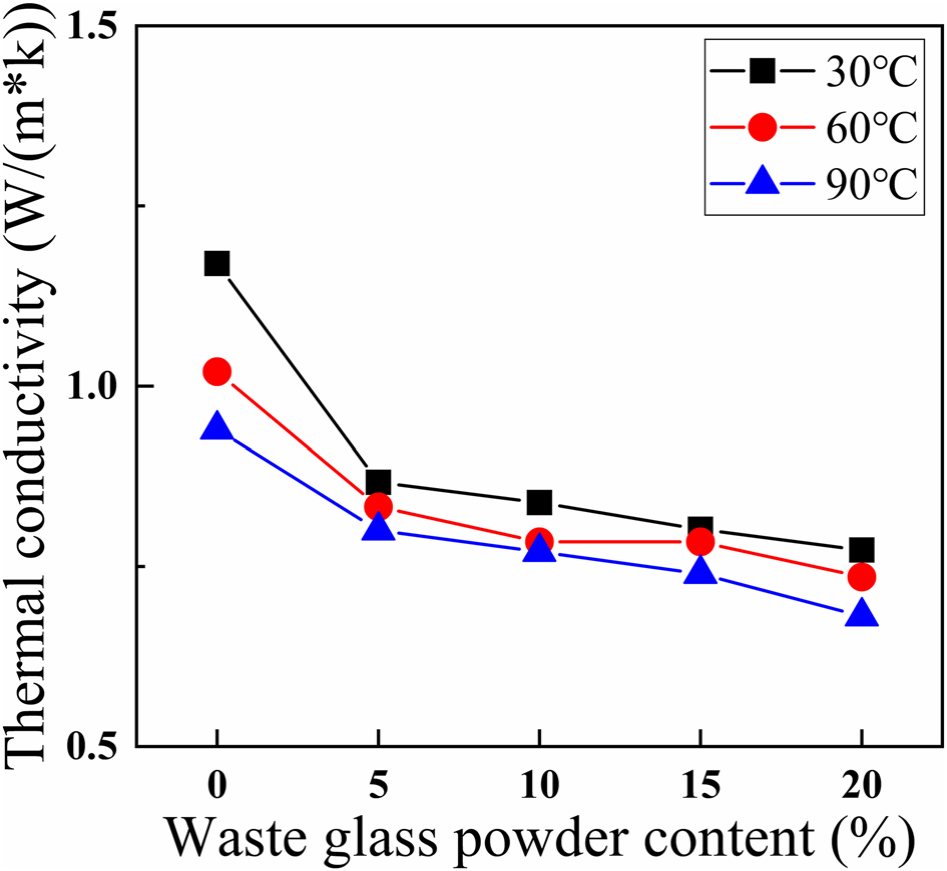
Thermal conductivity of waste glass powder cement.

Figure 5 clearly describes the thermal conductivity measured at 30 °C, 60 °C, and 90 °C for the cement paste with different waste glass powder contents. At 30 °C, the thermal conductivity of G0 and G4 were 1.1900 W/(m K) and 0.7728 W/(m K), respectively, with a decrease rate of 35.06%. At 60 °C, the thermal conductivity of G0 and G4 were 1.0300 W/(m K) and 0.7354 W/(m K), respectively, with a decrease rate of 28.60%. At 90 °C, the thermal conductivity of G0 and G4 were 0.9300 W/(m K) and 0.6810 W/(m K), respectively, with a decrease rate of 26.77%.

According to some previous researches, the thermal conductivity of 20% expanded perlite and 20% hollow bleached beads thermal insulation cements were reduced by 19.0% and 13.4%, respectively^9^. The thermal condutivity of cement with 25% limestone was reduced by 36.26%^38^. The thermal condutivity of cement with 50% blast furnace slag was reduced by 35%^40^. The thermal condutivity of cement paste with 30% Microwave Incinerated Rice Husk Ash was reduced 32.3%^41^. Compared with these thermal insulation materials, waste glass powder can significantly improve the thermal insulation performance of cement paste. The thermal insulation and mechanical properties of waste glass powder thermal insulation cement were better.

With the increase in the content of more waste glass powder, the thermal conductivity of insulating cement decreased. On the one hand, because glass is a complex and disordered substance with few free electrons, which is a poor conductor of heat; on the other hand, it is due to the volcanic ash reaction between waste glass powder and the hydration product of C_3_S, CH, generating C–S–H gel^42^. Due to the low crystallinity of C–S–H, the thermal conductivity of C–S–H gel is much lower than that of CH crystals^43^. The thermal conductivity of insulating cement decreased with rising temperature. This can be attributed to the irregular thermal movement of molecules within the cement paste at higher temperatures, which consumed some of the heat and led to a reduced thermal conductivity of the cement test block^44^. Additionally, the factors contributing to the decrease in thermal conductivity of the cement paste need to be further analyzed by thermogravimetric analysis tests.

### Thickening time

**Figure 6:**
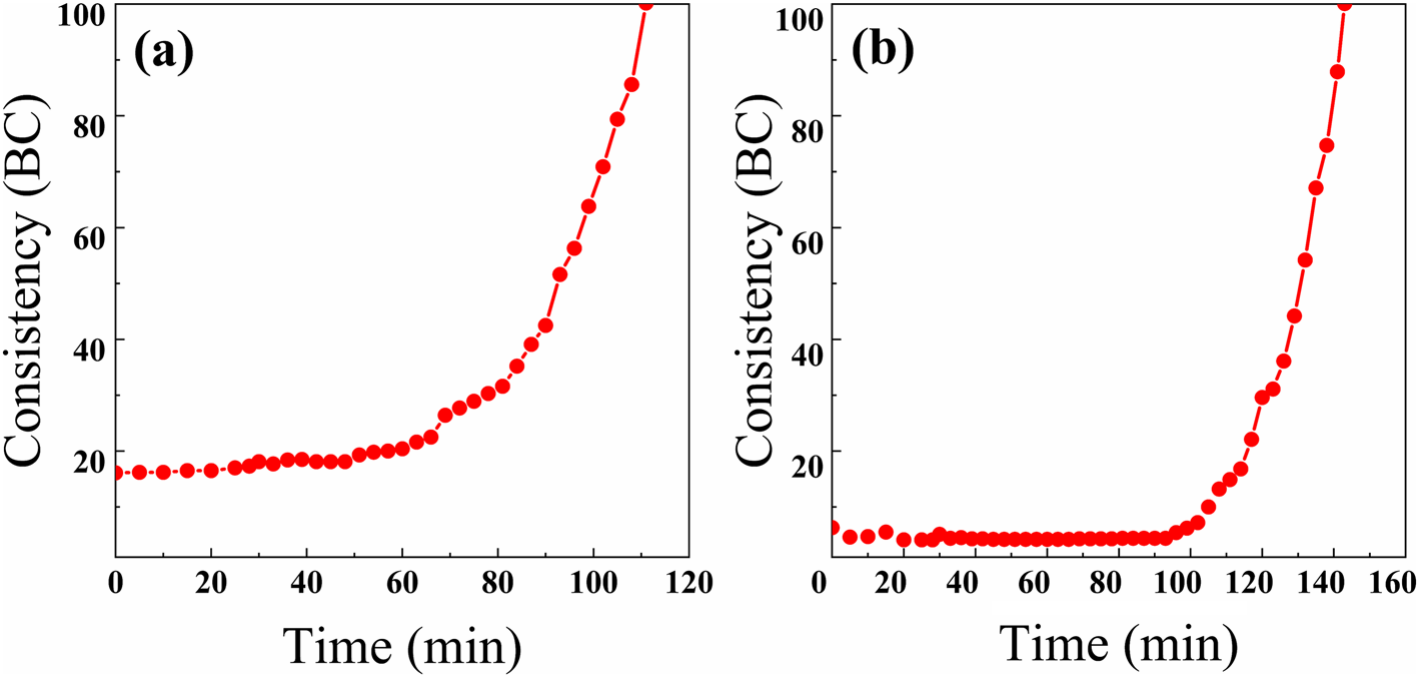
(**a**) Thickening time curve of pure cement paste; (**b**) Thickening time curve of 20% waste glass powder cement pasteThe thickening time curve of 20% waste glass powder cement paste with 0.1% water reducing agent and 0.01% retarder was shown in (**b**), the initial consistency was 6.3 BC, and it took 143 min to reach 100 BC, the thickening curve was at right angle. The consistency of the cement paste was maintained at 4.4 BC for 95 min and then increased rapidly to 100 BC.

The experimental conditions: the circulation temperature is 52 °C, the circulation pressure is 35.6 MPa, temperature and pressure rise time 28 min. The thickening time curve of the pure cement paste is depicted in Fig. 6a, the initial consistency was 16.1 BC, and it took 111 min to reach 100 BC, and the thickening curve showing a slowly increasing trend.

The thickening time of the cement paste is an important performance index for cementing construction and is the main basis for grasping and controlling the pumpable time of cement paste. Compared with the thickening curve of pure cement paste, the thickening curve of 20% waste glass powder cement slurry exhibited a long stable period, which was characterized with low initial consistency, better stability of the cement thickening, better pump ability, and long thickening time, facilitating the construction of pouring cement. Besides, the consistency increased rapidly in the later stage, which could enable the cement paste to solidify rapidly in a brief time and reduce the influence on the construction.

### XRD analysis

**Figure 7:**
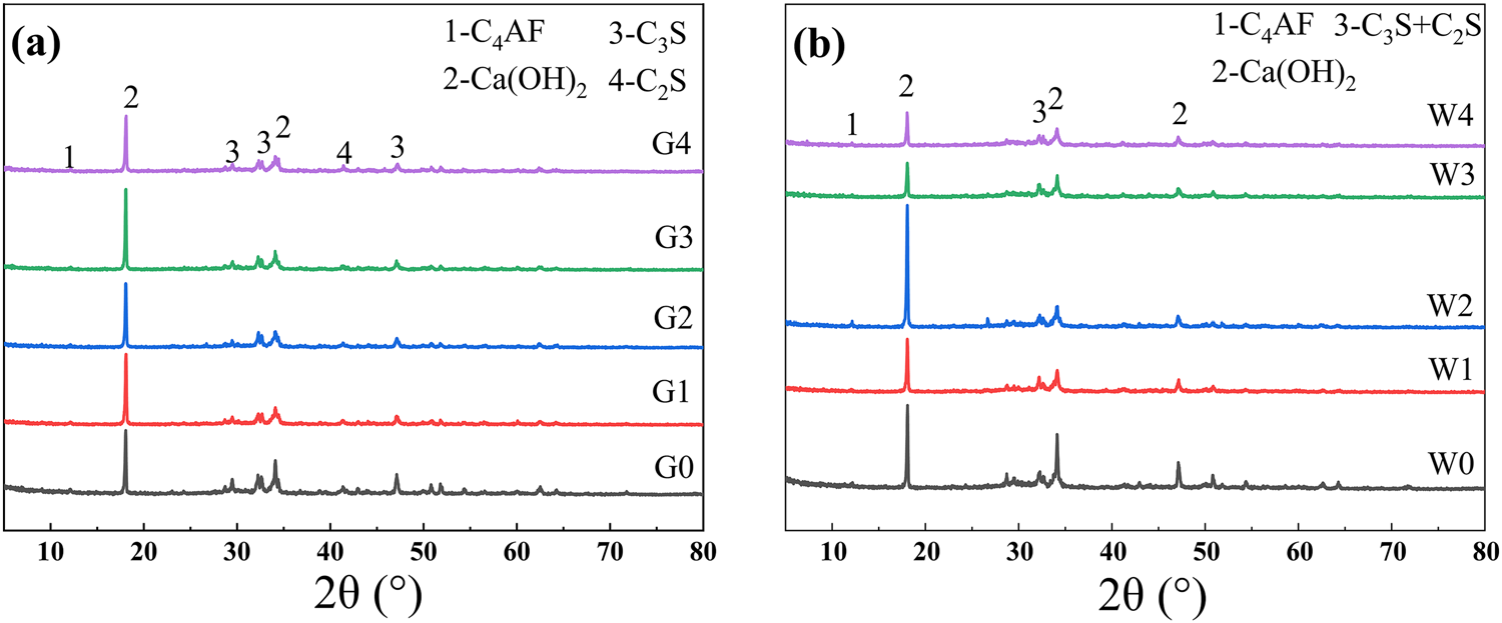
(**a**) XRD comparative diagram of G0-G4 cement paste; (**b**) XRD comparison diagram of W0-W4 cement paste.

Figure 7a and b show XRD diffraction patterns of G0-G4 cement paste and W0-W4 cement paste, respectively. As observed, no new hydration products were produced in the cement paste mixed with waste glass powder, when compared with pure cement paste. The main crystalline phase products are calcium hydroxide (CH), along with incompletely reacted tricalcium silicate (C_3_S) and brownmillerite (C_4_AF).

It is clear from Fig. 7a and b that the C_3_S diffraction peak near 32° decreased and the intensity of the CH diffraction peak near 18° and 34° decreased significantly with the increase in the content of waste glass powder, which was caused by this factor: the content of cement and C_3_S was reduced, leading to a decrease in the hydration product CH. Additionally, CH was decreased, the negative impact on the compressive strength of cement was reduced. Due to the high crystallinity of CH, its thermal conductivity is good. With the reduction of CH content, the thermal conductivity of the thermal insulation cement paste decreases^45^. Waste glass powder can increase the mechanical and thermal insulation properties of insulating cement.

Figure 7b demonstrates that the intensity of the C_3_S diffraction peak near 32° gradually decreased or even nearly disappeared as the content of waste glass powder increased, indicating a higher degree of C_3_S hydration in the cement paste at 60 °C. The diffraction peak of CH at 60 °C was significantly lower than that at 38 °C, which indicates that the amount of CH being consumed increases at high temperatures, and the negative impact on the compressive strength and thermal insulation properties of the insulating cement paste decreases.

### Thermo-gravimetric analysis

As can be seen from the above figure, the weight loss at 30–200 °C was mainly due to the evaporation of free water from the cement paste and the decomposition of C–S–H^46^, which corresponded to the first strong heat absorption peak of the DTG curve^47,48^. The weight loss at 400–500 °C was caused by the dehydration of hydroxyl groups in Ca(OH)_2_, which corresponded to the second strong endothermic peak of the DTG curve^48,49^. The third strong endothermic peak of the DTG curve, that is the weight loss between 550 and 700 °C, indicated CaO and CO_2_ overflow when CaCO_3_ began to decompose and decarburize^48,50^.

**Figure 8:**
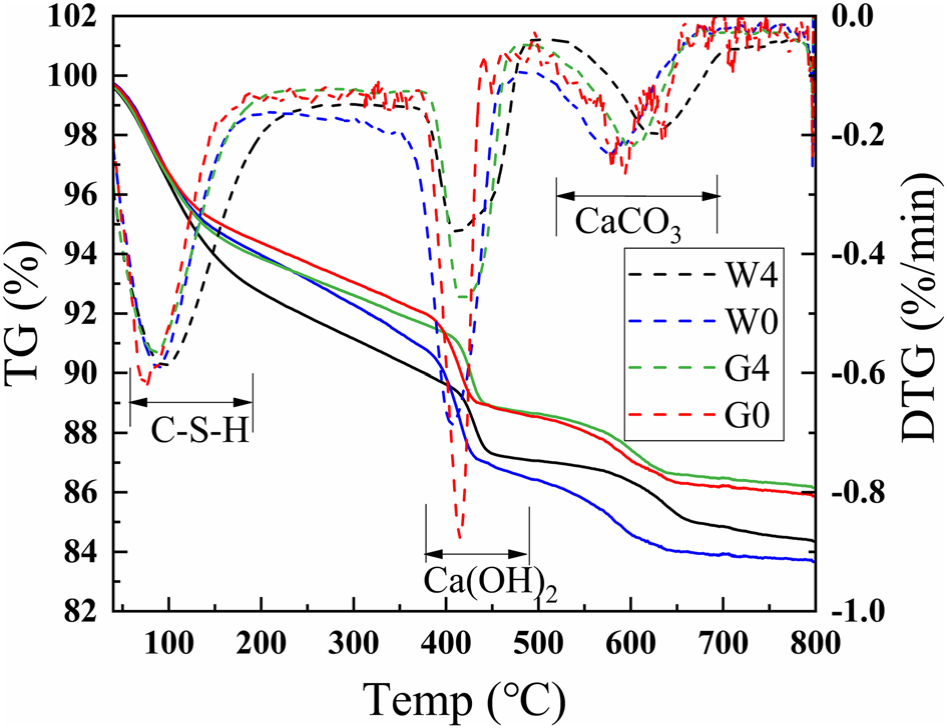
Thermal analysis curves of G0, G4, W0 and W4 cement paste.

Comparing W0 and W4, and G0 and G4, it can be seen that the intensity of strong exothermic peak of Ca(OH)_2_ of the thermal insulation cement with 20% of waste glass powder is obviously lower than that of the pure cement, on the one hand, it was because the addtion of waste glass powder led to the decrease of cement content, the amount of C_3_S was reduced, and the hydration product CH was reduced, and on the other hand, the SiO_2_ in the waste glass powder consumed part of the CH and generated C–S–H ^51^. By observed Fig. 8, it can be found that the weight loss of CH in W4 is slightly lower than that in G4, and W0 is slightly lower than that in G0, which demonstrates that the increase in temperature can promote the consumption of CH.CH has a negative impact on the compressive strength of the cement paste and the thermal insulation performance. That is say, the addition of waste glass powder can improve the mechanical properties and thermal insulation performance of cement paste.

Elevated temperatures may cause free water in the thermally conductive cement paste to evaporate and form pores. When heat is delivered to the pores, scattering occurs, resulting in a failure of heat transfer. The thermal conductivity of air is only 0.023 W/(m∙K), which is much lower than that of solids. Therefore, the thermal conductivity of cement paste decreases when the maintenance temperature increases.

### Mercury compression—pore structure

#### Porosity

**Figure 9:**
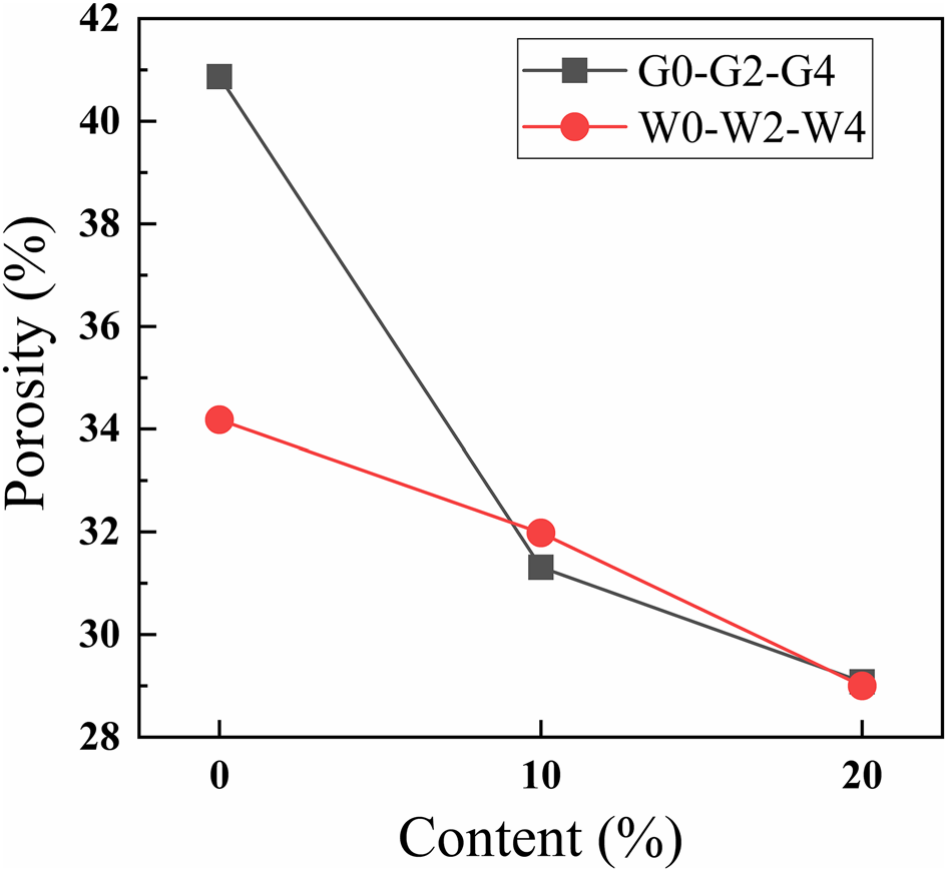
Porosity of cement paste with waste glass powder.

The porosity of waste glass powder insulation cement paste was evaluated by using the mercury-in-pressure (MIP) method and the results are shown in Fig. 9. The porosity of the thermal insulation cement decreased with the increase of the content of waste glass powder. The porosity of G0 was 40.87%, and the porosity of G2 and G4 decreased by 9.56% and 11.79%, respectively, compared with that of G0.

The porosity of G0 was 6.69% larger than that of W0, the porosity of W2 was 0.67% higher than that of G2, and that of W4 was 0.08% lower than that of G4, which indicates that the effect of temperature on the porosity of the insulating cement decreases with the increase in the amount of waste glass powder dosed.

The factors that lead to the reduction of the porosity of the thermal insulation cement are: (1). the micro-aggregate filling effect of the waste glass powder, the finer waste glass powder is filled in the voids of the cement paste, (2). the volcanic ash effect of the waste glass powder, the SiO_2_ in waste glass powder and CH in cement conduct secondary hydration to generate more C–S–H gel, which refines the pore structure of the thermal insulation cement paste. Waste glass powder can refine the pores, reduce the porosity of cement paste, and enhance the mechanical properties of thermal insulation cement paste.

### Pore size distribution

**Figure 10:**
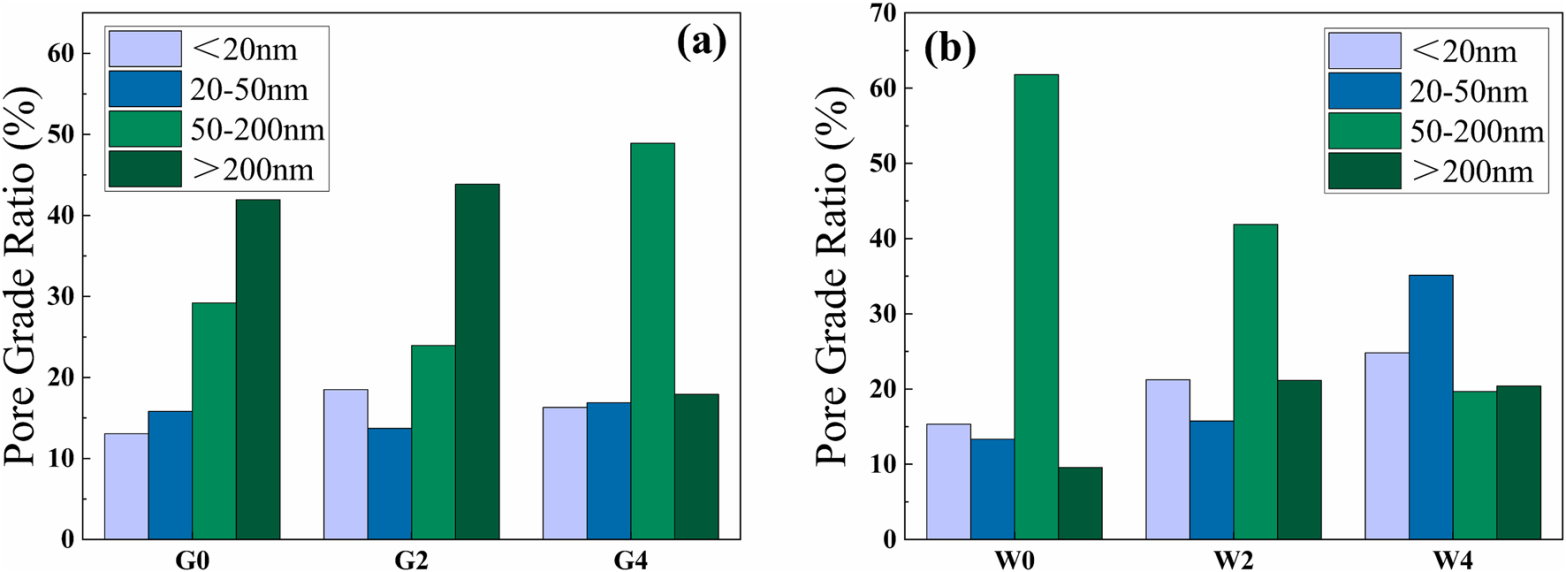
(**a**) Pore grade distribution of cement samples G0, G2 and G4; (**b**) pore grade distribution of cement samples W0, W2 and W4.

The pore grading method used in this paper divided the pores of cement paste into four classes: the harmless pores (pore size < 20 nm), the less harmful pores (pore size 20–50 nm), the harmful pores (pore size 50–200 nm) and the multi-harmful pores (pore size > 200 nm)^52^. This categorization helps to analyze the effect of waste glass powder on the pores of the insulting cement paste more deeply. Figure 10 shows the pore grading distribution of the insulating cement.

From Fig. 10a and b, the harmless pores, and the less harmful pores of the thermal insulation cement increased when the content of waste glass powder increased. After analysis, it can be concluded that the incorporation of waste glass powder can increased the amount of the harmless pores and the less harmful pores, reduced the harmful pores, and the effect of waste glass powder on the refinement of the pores of the thermal insulation cement paste was significant. The proportion of the harmless pores and the less harmful pores in W0, W2 and W4 are significantly higher than that in G0, G2 and G4, which indicates that the high temperature can promote the secondary hydration of cement, generate more C–S–H, refine the pore space, so that the further improvement of the pore structure of cement paste. In addition, micropores can enhance the heat transfer resistance of cement and decrease the thermal conductivity of the cement^53^. Waste glass powder can not only can refine the pores and increase the mechanical properties, but also can improve the thermal insulation performance of cement.

**Figure 11:**
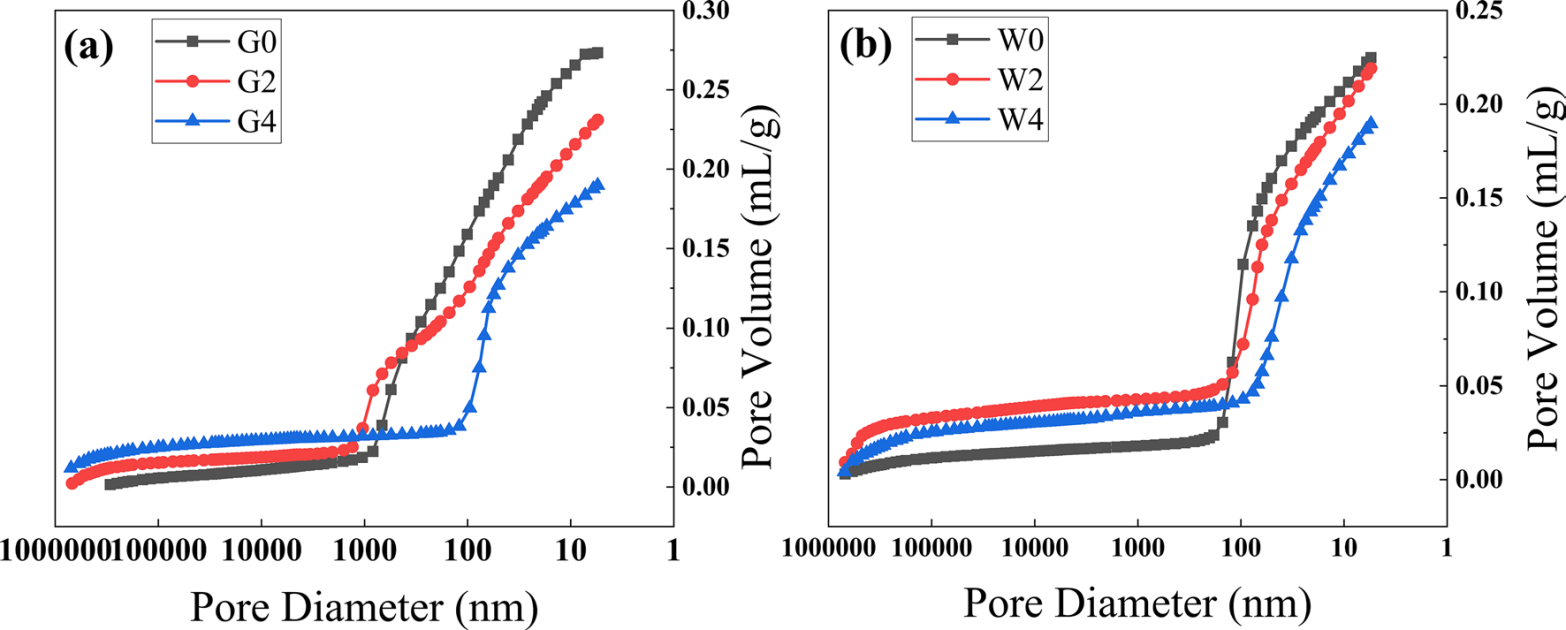
(**a**) Pore size distribution of G0, G2 and G4 cement samples; (**b**) pore size distribution of W0, W2 and W4 cement samples.

Figure 11 visually reflects the relationship between the pore volume and the pore size distribution of the insulating cement. As shown in Fig. 11a, in terms of the pore volume and the pore size, the pore volume of G4 is significantly reduced compared with that of G0 and G2, and these pore volume reductions start from a critical aperture: 850 nm for G0, 1050 nm for G2, and 180 nm for G4. Figure 11b shows a smaller difference in the pore volume and the pore size of W0, W2, and W4, and the critical pore size for the pore volume reduction was 120 nm for W0, 95 nm for W2 and 77 nm for W4, respectively.

In summary, the pore volume of the cement paste was refined, and the porosity decreased with the increase of temperature and the content of waste glass powder. The pore evolution process proved to be crucial for improving thermal insulation properties and compressive strength of cement paste.

#### Microscopic morphology analysis

**Figure 12:**
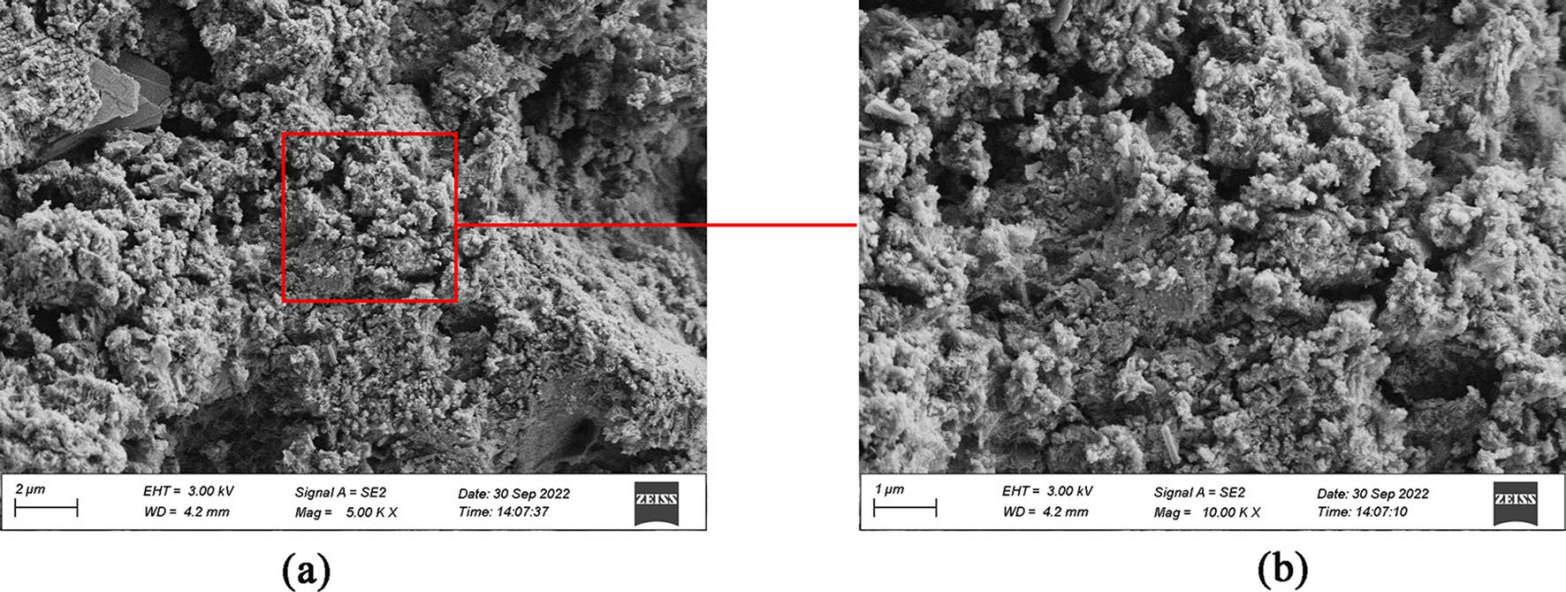
(**a**) G0 cement paste (5K), (**b**) G0 cement paste (10K).

Figure 12a and b show the 5K image and the 10K image of G0 cement paste, respectively. As observed, the cement paste structure was irregularly arranged with more obvious pores and cracks, as well as the presence of discontinuously distributed granular material. The overall structure was loose and uncompact.

**Figure 13:**
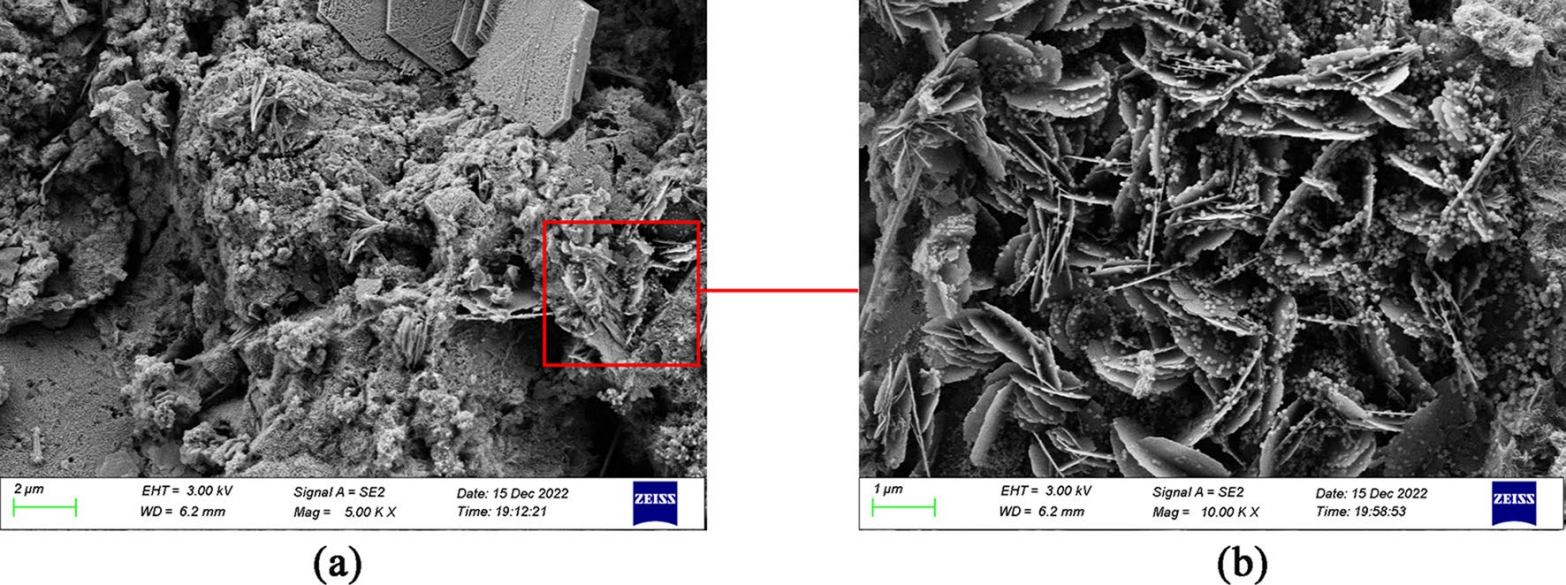
(**a**) G4 cement paste (5K), (**b**) G4 cement paste (10K).

Figure 13a and b describe the 5K image and the 10K image of G4 cement paste, respectively. Compared with G0, the G4 cement paste has an irregularly arranged structure with smaller pores and higher density. Furthermore, the fibrous and layered C–S–H can be observed in G4 cement paste, and there are many discontinuous particles distributed on the layered C–S–H, which increase the thermal resistance of the cement.

**Figure 14:**
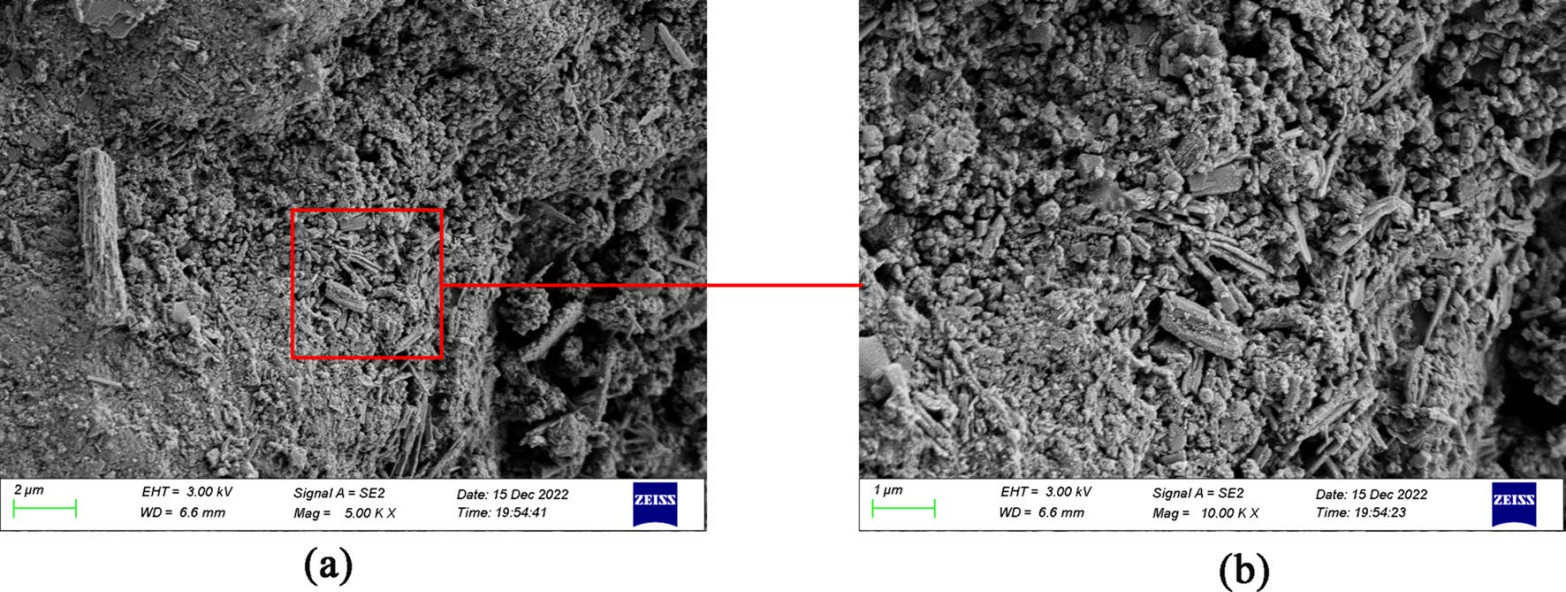
(**a**) W0 cement paste (5K), (**b**) W0 cement paste (10K).

Figure 14a and b display the 5K image and the 10K image of W0 cement paste, respectively. It has a relatively dense overall structure, with visible pores and unevenly distributed.

**Figure 15:**
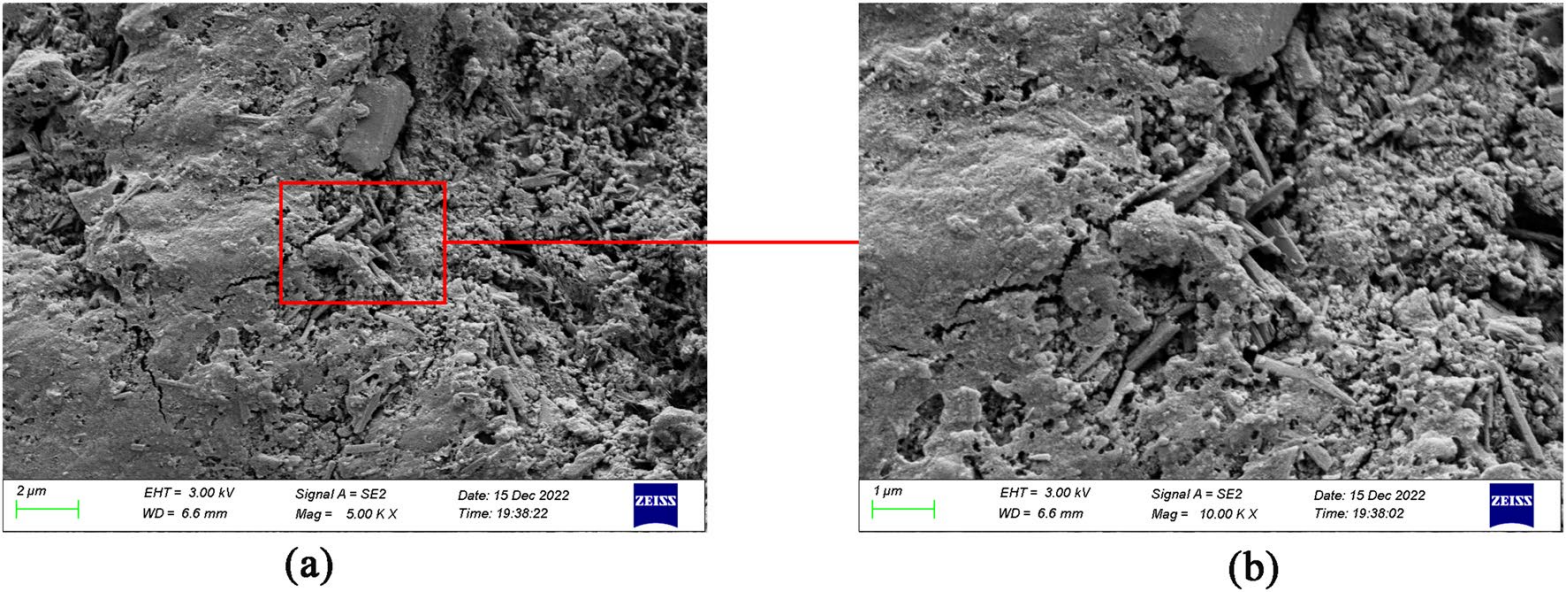
(**a**) W4 cement paste (5K), (**b**) W4 cement paste (10K).

Figure 15a and b depict the 5K image and 10K image of W4 cement paste, respectively. The overall structure of W4 is much denser. The large amount of C–S–H intertwines with each other, transforming the larger harmful pores into smaller harmful and harmless pores, and even leading to the disappearance of the pores.

In general, with the increase in the content of waste glass powder and the raise in the curing temperature, the overall compactness of the cement pastes increased, and the compressive strength of the cement is enhanced. In addition, it can be observed from the figure that, with the increase of the content of waste glass powder, more C–S–H is generated by the volcanic ash effect of SiO_2_ with CH.

Solid heat transfer in inorganic nonmetallic cement materials is mainly accomplished by low-frequency phonons from lattice oscillations. C–S–H is a kind of gel substance with low crystallinity, and its thermal conductivity is much lower than that of CH crystals. C–S–H is disordered in atomic reactions, many not closely distributed fibrous C–S–H and decreasing continuum particles improve the thermal resistance of the cement paste. Secondly, the uniformly distributed small pores in the sample, the waste glass and the cement paste stacked on top of each other form a disordered high thermal resistance structure, and these structures make the interior of the sample more complex, increase the probability of various defects appearing, and cause the phonon scattering to be continuously enhanced, so the waste glass powder can reduce the thermal conductivity of the cement paste, and improve the thermal insulation property of the cement paste.

## Conclusion and outlook

1. Aiming at the neglect of mechanical properties in the study of thermal insulation cement in the upper part of geothermal wells, this study blends waste glass powder as thermal insulation material into cement to prepare thermal insulation cement, and analyzes the performance of waste glass powder thermal insulation cement by using macroscopic test and microanalysis, and the main conclusions are as follows: From the systematic test results, in the content of 0–20% waste glass powder, the optimal mixing ratio of thermal insulation cement is 20% waste glass powder + 80% G grade oil well cement.
2. The mechanical properties, thermal conductivity, and thickening characteristics of thermal insulation cement with 20% waste glass powder are as follows: the compressive strength of 1 d of curing at 38 °C and 60 °C was enhanced by 16.10% and 39.08%, respectively. The thermal conductivity of the cement maintained at room temperature for 28 d was reduced by 35.06%, 28.60% and 26.77% at 30 °C, 60 °C and 90 °C, respectively. And thermal insulation cement with 20% waste glass powder at 52 °C, the thickening characteristics of right-angle thickening, the process of no false solidification, flash condensation or settlement phenomenon occurs.
3. According to the microscopic interpretation of TG, MIP and SEM, the macroscopic law of waste glass powder thermal insulation cement, due to the microaggregate effect of waste glass powder and the SiO_2_ in waste glass powder can be secondary hydration reaction with cement hydration product CH to generate more C–S–H gel, attached to the cement hydration product, which can effectively reduce the porosity of the thermal insulation cement and play a role in the role of refinement of the pore structure, the less-harmful and the harmless closed pores increased which can and enhance the compressive strength of thermal insulation cement.
4. Combined with XRD, TG and SEM micro-analysis of the thermal insulation mechanism of thermal insulation cement with waste glass powder. Waste glass powder with disordered and complex pore structure makes the internal structure of the cement more complex increasing the high thermal resistance structure of the cement, which enhances the phonon scattering of the cement, leads the thermal conductivity decreased and thermal insulation performance increased.
5. This research can change the status quo of insufficient mechanical properties of thermal insulation cement in geothermal well, so that the thermal insulation cement can meet the thermal insulation performance while ensuring that its mechanical properties do not decrease, increase the service life of geothermal wells, and have important engineering value and significance for improving the thermal energy extraction rate of geothermal wells.

Currently, the cost of many thermal insulation materials is high, and the use of waste glass powder instead of other thermal insulation materials can effectively save the purchase cost of raw materials. The application of waste glass powder as thermal insulation material can expand the application potential of waste glass powder, and reduce waste emissions, the pressure on landfills, the pollution of soil and groundwater resources and make an important contribution to environmental protection and resource saving, and the amount of cement and effectively improve the thermal insulation properties of oil well cement, as well as reduce the CO_2_ produced emissions. The application of waste glass powder as thermal insulation material to prepare thermal insulation cement in the middle and upper section of the geothermal wells can effectively reduce the heat transfer of geothermal energy to the stratum, reduce heat loss and improve the extraction rate of geothermal energy. The results of this research are of great significance to improve the heat extraction efficiency of geothermal energy. Further consideration should be given to the long-term stability and durability of thermal insulation cement under geothermal conditions in future research to ensure the suitability and practicality of the material.

## Data Availability

All data generated or analyzed during this study are included in this published article.
